# The use of OCT to detect signs of intracranial hypertension in patients with sagittal suture synostosis: Reference values and correlations

**DOI:** 10.1007/s00381-022-05598-1

**Published:** 2022-08-16

**Authors:** Stephanie D. C. van de Beeten, Wishal D. Ramdas, Sumin Yang, Sjoukje E. Loudon, Bianca K. den Ottelander, Dimitris Rizopoulos, Marie-Lise C. van Veelen, Irene M. J. Mathijssen

**Affiliations:** 1 Dutch Craniofacial Center, Department of Plastic and Reconstructive Surgery and Hand Surgery, Erasmus MC–Sophia Children’s Hospital, University Medical Center Rotterdam, Room EE-1591, Postbus 2040, 3000 CA, Wytemaweg 80, 2015 CN Rotterdam, The Netherlands; 2 Department of Ophthalmology, Erasmus MC–Sophia Children’s Hospital, University Medical Center Rotterdam, Postbus 2040, 3000 CA, Rotterdam, The Netherlands; 3 Department of Biostatistics, Erasmus MC, University Medical Center Rotterdam, Postbus 2040, 3000 CA, Rotterdam, The Netherlands; 4 Department of Neurosurgery, Erasmus MC–Sophia Children’s Hospital, University Medical Center Rotterdam, Room SK-1204, Postbus 2040, 3000 CA, Rotterdam, The Netherlands

**Keywords:** Craniosynostosis, Intracranial pressure, Papilledema, Optical coherence tomography

## Abstract

**Purpose:**

To obtain pediatric normative reference values and determine whether optical coherence tomography (OCT) corresponds better with clinical signs of intracranial hypertension (ICH) compared to the traditional screening method fundoscopy in a large cohort of one type of single suture craniosynostosis.

**Methods:**

Control subjects without optic nerve diseases and isolated sagittal synostosis patients aged 3–10 years who underwent fundoscopy and OCT were included in this prospective cohort study. Normative reference values were obtained through bootstrap analysis. Main outcome was the association between peripapillary total retinal thickness (TRT) and total retinal volume (TRV) and appearance on fundoscopy. Signs and symptoms suggestive of ICH, including skull growth arrest, fingerprinting, and headache, were scored.

**Results:**

Sixty-four healthy controls and 93 isolated sagittal synostosis patients were included. Normative cut-off values for mean TRT are < 256 μm and > 504 μm and for mean TRV < 0.21 mm^3^ and > 0.39 mm^3^. TRT was increased in 16 (17%) and TRV in 15 (16%) of 93 patients, compared to only 4 patients with papilledema on fundoscopy (4%). Both parameters were associated with papilledema on fundoscopy (OR = 16.7, *p* = 0.02, and OR = 18.2, *p* = 0.01). Skull growth arrest was significantly associated with abnormal OCT parameters (OR = 13.65, *p* < 0.01).

**Conclusions:**

The established cut-off points can be applied to screen for ICH in pediatrics. The present study detected abnormalities with OCT more frequent than with fundoscopy, which were associated with skull growth arrest. Therefore, a combination of OCT, fundoscopy, and skull growth arrest can improve clinical decision-making in craniosynostosis.

## Background

One of the challenges in treating patients with craniosynostosis is the detection of intracranial hypertension (ICH). In anticipation of better alternatives, papilledema on fundoscopy is the most used screening tool in the clinic. However, this subjective measurement often detects ICH in a late phase and lacks the ability of precise follow-up [1]. There is a need for an objective and quantitative measurement for ICH which can be combined with other clinical data that correlate with ICH, such as skull growth arrest, to optimize clinical decision-making. With ICH, impaired axoplasmic flow can result in structural changes of the retina [2]. With optical coherence tomography (OCT), changes in the retina can be detected with a high degree of precision. This noninvasive imaging technique facilitates an objective assessment of multiple layers of the retina: peripapillary total retinal thickness (TRT), peripapillary total retinal volume (TRV), and retinal nerve fiber layer thickness (RNFL). These highly reproducible measurements make an accurate follow-up of individual patients possible.

Prior OCT studies have reported that TRT, TRV, and RNFL are increased in case of ICH [3–9], with TRT and TRV shown to be more accurate in monitoring and diagnosing papilledema, especially in the pediatric group [10]. Literature on OCT in craniosynostosis is scarce, but all conclude to have high potential as a quantative screening method to detect papilledema. Our previous pilot study demonstrates that OCT in craniosynostosis is feasible from the age of 3 years [3]. In absence of normative data, Driessen et al. compared the TRT of patients with and without papilledema on fundoscopy and found an increase in TRT in patients with papilledema [3]. Dagi et al. [11] described similar results for the RNFL. More recently, Swanson et al. [8] reported a sensitivity of 89% and specificity of 62% for detecting invasively measured ICH when combining two manually measured OCT parameters in young patients with craniosynostosis.

However, OCT measurements differ per device and segmentation algorithm [12, 13]. Normative data on TRT and TRV automatically obtained by Spectralis SD-OCT (Heidelberg Engineering, Dossenheim, Germany) is lacking among the pediatric population. Therefore, we first aim to establish normative reference values to facilitate the use of OCT as a screening tool in children. Secondly, we aim to determine whether OCT corresponds better with clinical signs of ICH compared to the traditional screening method fundoscopy in a cohort of one type of single suture craniosynostosis.

## Methods

### Participants

In this prospective cohort study, healthy control subjects were recruited in 2014 at our Department of Ophthalmology. Non-syndromic sagittal synostosis patients presenting to the Dutch Craniofacial Center were recruited between April 2010 and February 2019.

### Healthy control cohort

Children without a condition affecting the retina thickness, aged 4–10 years, were enrolled if they had dilated pupils because of the clinical examination scheduled at the Department of Ophthalmology. Children with high hyperopia (≥ + 4 D) and high myopia (≤ − 2.5 D) were excluded. All children underwent ophthalmic examination including cycloplegic refractive error (RE) measurement, fundoscopy, and an OCT scan. RE was measured in diopters (D) 30 min after instillation of cyclopentolate 1% using an autorefractor (Topcon, Tokyo Optical Co., Japan). RE between − 2.5D and + 4D is defined as mild.

### Sagittal synostosis cohort

**Fig. 1:**
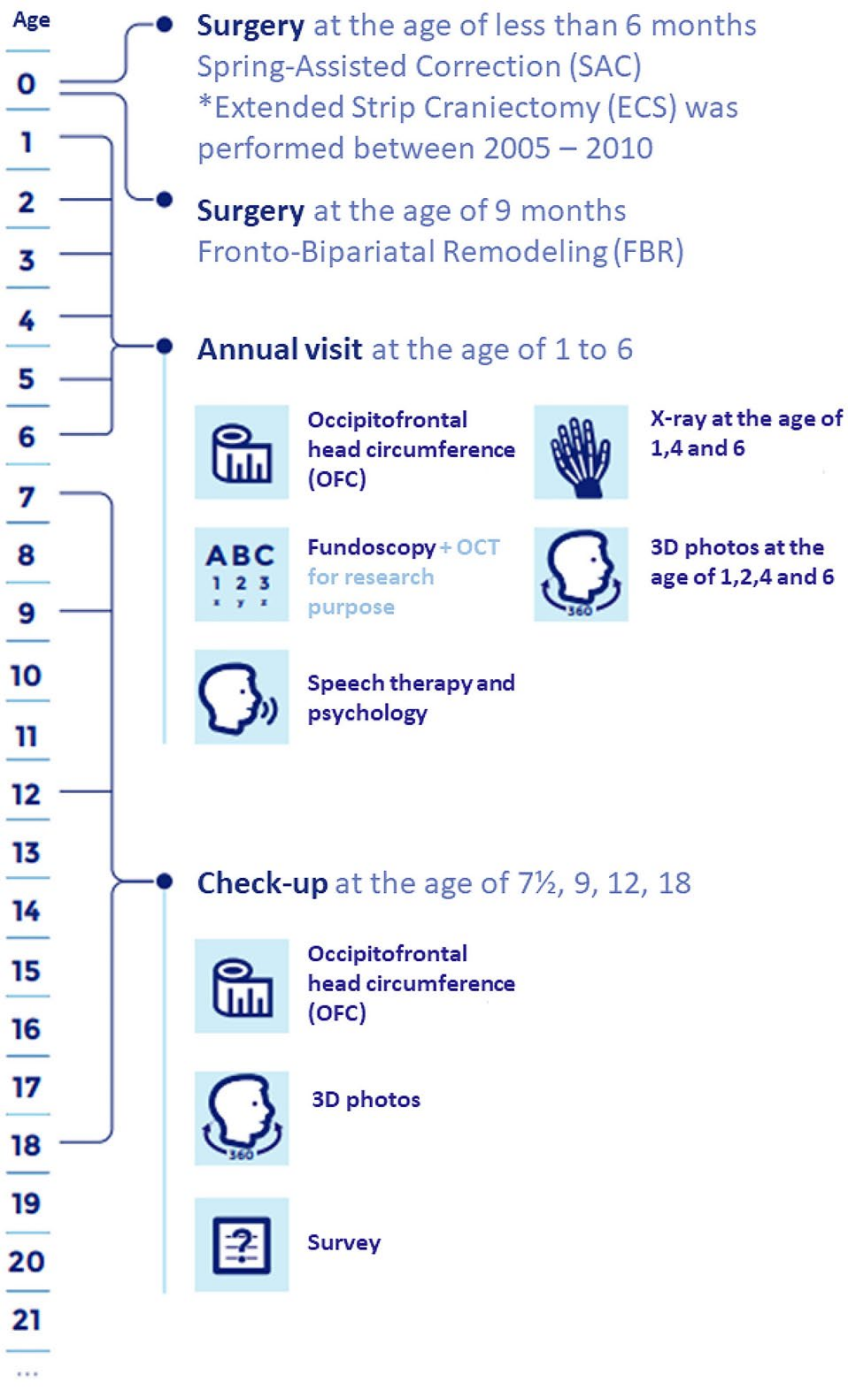
Our centers follow-up protocol for sagittal synostosis patients

Isolated sagittal synostosis patients, aged 3–10 years presenting at our center for a follow-up appointment involving fundoscopy, were included. Data was collected prospectively according to our follow-up protocol shown in Fig. 1.

### Sagittal synostosis — OFC

OFC is a method that has shown to reliably correlate to intracranial volume (ICV) [14]. A stagnation or decline of the OFC curve can predict the onset of ICH [15, 16]. The OFC was measured in centimeters and converted into standard deviations according to the Dutch National Standards. OFC trajectories, 1 year post-surgery until the day of the OCT scan, were analyzed by two surgeons and skull growth arrest was defined as a SD fall of 0.5 or more from baseline over 2 years.

### Sagittal synostosis — fingerprinting

**Fig. 2:**
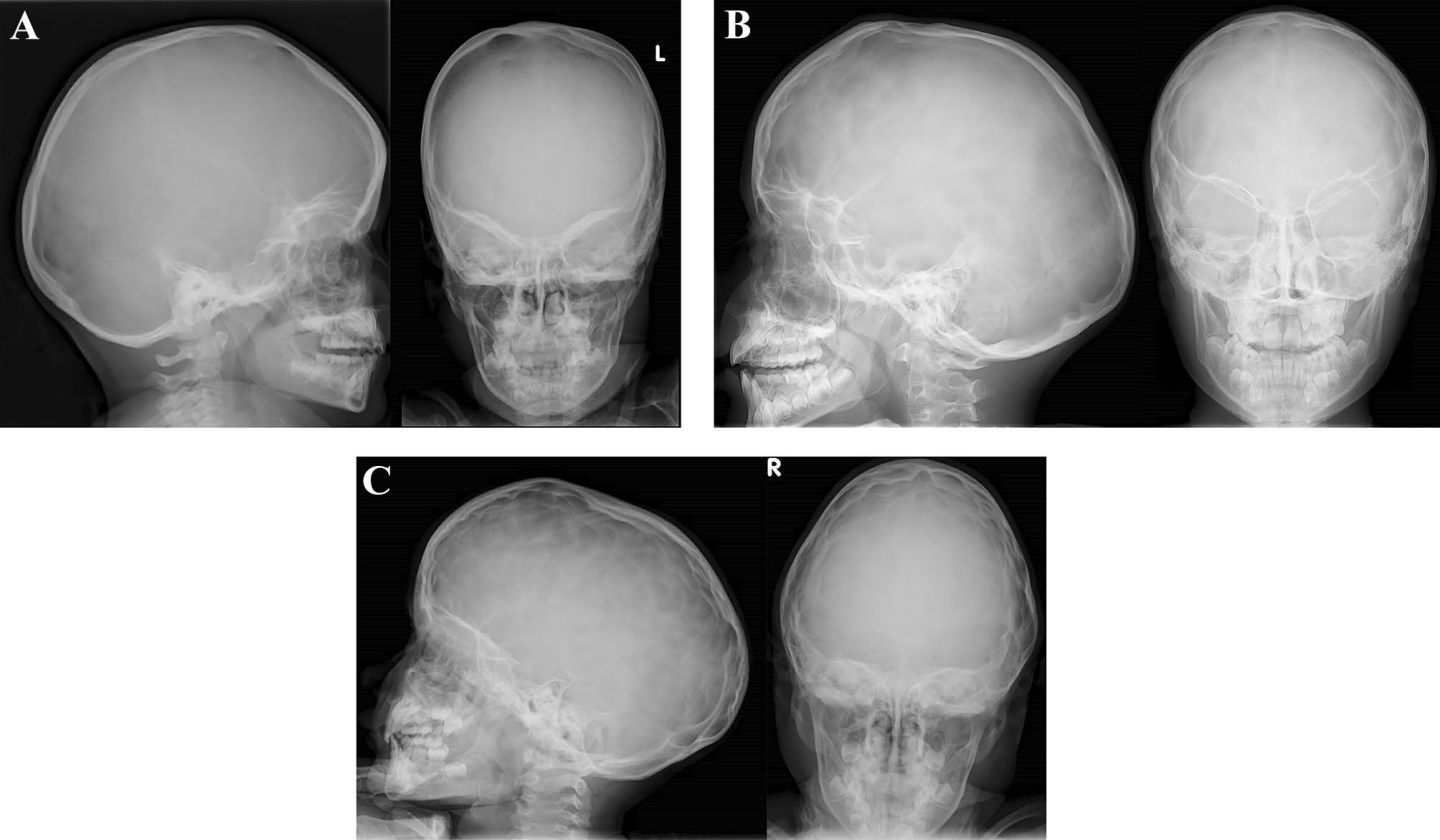
Fingerprinting on skull X-ray. **A** Normal skul X-ray, **B** minimal fingerprinting, **C** extensive fingerprinting

Fingerprinting can be a sign of ICH [17–20]. For this study, postoperative skull radiographs within 1 year of the OCT scan were included. Occurrence of diffuse fingerprinting was scored by two blinded observers on a 3-point scale: none (0) — minimal (1) — extensive (2) and was converted into a two-point scale. In this way, only extensive fingerprinting was scored as abnormal (0 = normal/minimal, 1 = extensive fingerprinting) (Fig. 2). When the scores of the two observers did not correspond, the observers re-evaluated the radiographs together to reach consensus. There was a high interrater reliability (kappa 0.90, *p* < 0.001, 95% CI 0.77–1.03).

### Fundoscopy

Both groups underwent fundoscopy and OCT on the same day. An ophthalmologist performed fundoscopy under mydriasis with phenylephrine tropicamide in patients with sagittal synostosis. Pupils of healthy children were already dilated due to the RE measurement. Papilledema is defined as edema of the optic disc or blurring of the optic disc margins and needs to be differentiated from pseudopapilledema caused by hyperopic crowded nerve or optic disc drusn. Patients suspected of hyperopia underwent RE measurement, and B-scan ultrasound (Avisio, Quantel Medical, Clermont-Ferrand, France) was performed to confirm drusen. In case of at least two consecutive deviating funduscopic examinations, patients were stratisfied in the papilledema group.

### Spectral-domain optical coherence tomography

**Fig. 3:**
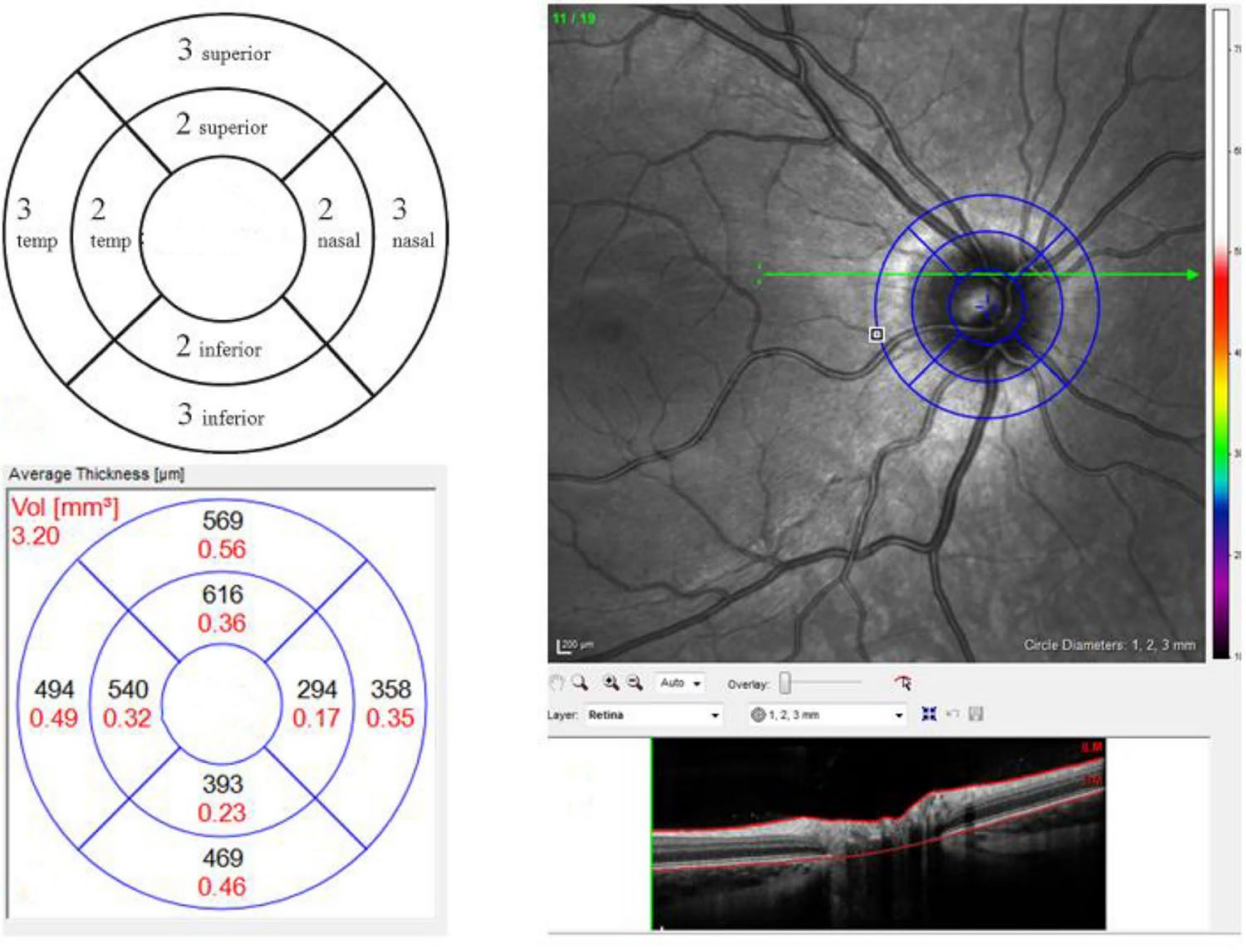
Volume scan. The volume scan provides both retinal thickness and retinal volume data per quadrant of the optic disc. 2A The circle is divided in inner and outer circle at a distance of 2 and 3 mm from the center with 2 × 4 equal quadrants (superior, inferior, nasal, and temporal). 2B In order to obtain the total retinal thickness and total retinal volume, a circular chart is positioned over the optic disc. 2C The mean retinal thickness in μm is presented in black and total retinal volume in mm^3^ is presented in red. In this study, the mean of all the 8 quadrants provides the total retinal thickness and total retinal volume. The mean of the 4 outer quadrants (3-superior, 3-inferior, 3-nasal, and 3-temporal) represents the outer total retinal thickness and outer total retinal volume. The inner circle is calculated in the same way

Imaging was performed using the Spectralis OCT scanner (Heidelberg Engineering, Dossenheim, Germany). In all children, the TRT and TRV of the optic disc were analyzed. The TRT and TRV were measured on a volume scan consisting of 19 horizontal sections over an area of 20 × 15° (Fig. 3). Internal or external fixation was used to center optic disc, after which the retinal image was focused to optimize the quality of the scan. The interlimiting membrane (ILM) and Bruch’s membrane, the reference layers for the TRT, are automatically detected by SD-OCT segmentation algorithms. In order to determine the TRT and TRV, a circular chart was positioned over the exact center of the optic disc. The diameters of the circle were 1, 2, and 3 mm, resulting in 2 × 4 equal quadrants (superior, inferior, nasal, and temporal; Fig. 3). The mean of all eight areas was calculated, resulting in a TRT and TRV. OCT scans in which less than 75% of the areas were available were excluded.

### Statistical analysis

Statistical analysis was performed in R statistical software (version 4.1.3). Paired *t*-test was used to compare OCT parameters between right and left eyes. Since there was no significant difference between the two eyes, only one eye of each participant was included in further analyses. For normative data, the right eye was used unless the scan of the right eye was of low quality or was excluded based on RE, in accordance with the majority of ophthalmology studies [21, 22]. The selection of the right eye is considered statistically valid under the assumption that normative values do not favor a particular eye and can therefore be generalized [23]. In sagittal synostosis patients, the eye with the highest TRT was selected, as papilledema can occur unilateral in these patients. OCT data were expressed as median (interquartile range (IQR)). Shapiro–Wilk test assumed normal distribution for TRT data in normal pediatrics. We applied a non-parametric bootstrap method to compute reference intervals (RIs) with corresponding 95% confidence intervals (CIs) for TRT and TRV. The CIs are constructed for the lower and upper end points, to determine the precision of the RIs. A quantile regression analysis was performed to assess the effect of age, gender, and RE on the OCT parameters. To meet the assumptions, a Wilcoxon rank-sum test was performed to compare the OCT values of the right eye between the control group and sagittal synostosis group. Next, using cut-off values derived from the control cohort, primary outcome measures TRT and TRV were transformed in categorical variables and its association with fundoscopy was analyzed by using Fisher’s exact test. Secondary outcomes comprised the association with clinical symptoms of ICH and sub-analysis within the OCT parameters. The interrater reliability of the reviewers was assessed for fingerprinting with the Cohen kappa (*κ*). *p*-values of < 0.05 were considered statistically significant. Our primary outcome measures did not involve multiple testing, and therefore, Bonferroni correction was not required. Secondary outcomes were not corrected for multiple testing.

## Results

### Participant

One hundred sixty-one children were included: 67 healthy controls and 94 isolated sagittal synostosis patients. Based on the RE measurement performed in the control group, 3 children with high hyperopia were excluded. In the sagittal synostosis group, one patient did not have a reliable scan, resulting in 64 controls and 93 isolated sagittal synostosis patients included in this study (*N* = 157 children). The control cohort comprised 28 males (44%) and the scaphocephaly cohort 73 (78%) (Fisher’s exact, *p* < 0.001). The median age was 8.4 years (IQR 6.8–9.7, range 4.4–10.8) for healthy controls and 5.2 years (IQR 4.3–6.3, range 2.8–9.9; *p* < 0.001) for sagittal synostosis patients. Following treatment protocol, 89 sagittal synostosis patients underwent skull surgery, 4 patients did not undergo a surgical correction due to a late referral to the clinic without the presentation of ICH.

### Normative data: age, gender, and refractive error

All healthy controls had normal funduscopic examinations. The median TRT was 377.9 μm (IQR 337.80–404.38) and the median TRV was 0.29 mm^3^ (IQR 0.27–0.31). Effects of age, gender, and RE are presented in Table 1.

**Table 1:** Effect of age, gender, and mild refractive error on peripapillary TRT and TRV in healthy children

**Variable**	**TRT change (μm)**	**95% CI**	***p***	**TRV change (mm**^**3**^**)**	**95% CI**	***p***
**Age (year)**	− 0.85	− 13.32–7.73	0.88	− 0.002	− 0.012–0.003	0.63
**Gender (male)**	23.54	− 8.10–56.77	0.18	0.013	− 0.012–0.03	0.26
**Mild refractive error (D)**	2.06	− 6.79–13.20	0.68	0	− 0.007–0.007	0.91

The right eye is taken

*D* diopters, *TRT* total retinal thickness, *TRV* total retinal volume; mild refractive error: between − 2.5D and + 4D

### Normative data: cut-off points

The TRT and TRV normative reference ranges for the control group are shown in Table 2.

**Table 2:** Normative reference ranges for the mean peripapillary TRT and TRV in children aged 4–10 years

**Parameter**	**Lower RI**	*95% CI*		***Upper RI***	*95% CI*		*Missing*
		*Lower*	*Upper*		*Lower*	*Upper*	*N*
TRT	**256.0**	212.0	289.6	**503.8**	500.0	556.2	0
Outer ring	**313.5**	294.8	326.7	**443.9**	424.5	478.0	3
Inner ring	**198.9**	130.5	253.1	**565.1**	555.2	636.8	2
Superior quadrant	**198.9**	134.7	253.1	**565.1**	555.2	640.4	1
3-superior	**322.8**	291.0	339.6	**462.0**	459.0	476.8	1
2-superior	**180.1**	90.4	223.3	**573.6**	569.3	626.4	0
Nasal quadrant	**248.8**	195.6	289.0	**551.2**	510.4	647.4	2
3-nasal	**288.3**	269.6	294.7	**453.5**	392.0	514.4	2
2-nasal	**198.6**	110.6	262.2	**662.4**	655.8	788.8	1
Inferior quadrant	**285.3**	246.6	309.6	**563.3**	547.1	600.4	1
3-inferior	**337.4**	325.3	341.8	**506.6**	497.3	546.8	0
2-inferior	**233.4**	176.4	277.8	**641.8**	641.8	739.6	1
Temporal quadrant	**218.7**	181.6	238.4	**429.0**	409.0	497.1	0
3-temporal	**287.5**	277.0	295.0	**385.4**	364.8	405.3	0
2-temporal	**149.9**	98.8	181.8	**477.0**	462.0	579.3	0
TRV	**0.21**	0.17	0.22	**0.39**	0.38	0.42	0
Outer ring	**0.31**	0.29	0.32	**0.44**	0.42	0.47	3
Inner ring	**0.12**	0.08	0.15	**0.33**	0.33	0.38	2
Superior quadrant	**0.21**	0.16	0.23	**0.40**	0.39	0.42	1
3-superior	**0.32**	0.29	0.34	**0.45**	0.45	0.47	1
2-superior	**0.11**	0.05	0.13	**0.34**	0.34	0.37	0
Nasal quadrant	**0.21**	0.17	0.23	**0.41**	0.38	0.48	2
3-nasal	**0.28**	0.27	0.29	**0.45**	0.38	0.51	2
2-nasal	**0.12**	0.06	0.15	**0.39**	0.39	0.46	1
Inferior quadrant	**0.25**	0.21	0.25	**0.43**	0.42	0.46	1
3-inferior	**0.33**	0.32	0.33	**0.50**	0.49	0.54	0
2-inferior	**0.14**	0.11	0.17	**0.39**	0.38	0.44	01
Temporal quadrant	**0.19**	0.16	0.20	**0.33**	0.31	0.38	0
3-inferior	**0.28**	0.27	0.30	**0.38**	0.36	0.41	0
2-inferior	**0.09**	0.06	0.11	**0.28**	0.28	0.35	0

TRT in μm and TRV in mm^3^. The right eye is taken

*RI* reference interval, *CI* confidence interval, *TRT* total retinal thickness, *TRV* total retinal volume

### Sagittal synostosis cohort: OCT parameters correlated with fundoscopy and clinical signs

A median TRT of 403.8 μm (IQR 368.6–453.4) and median TRV of 0.31 mm^3^ (IQR 0.29–0.34) were found in the sagittal synostosis patients, both were increased compared to the control group (Wilcoxon rank-sum test, for both TRT and TRV *p* < 0.001). In four patients (4.3%), papilledema was detected with fundoscopy.

**Fig. 4:**
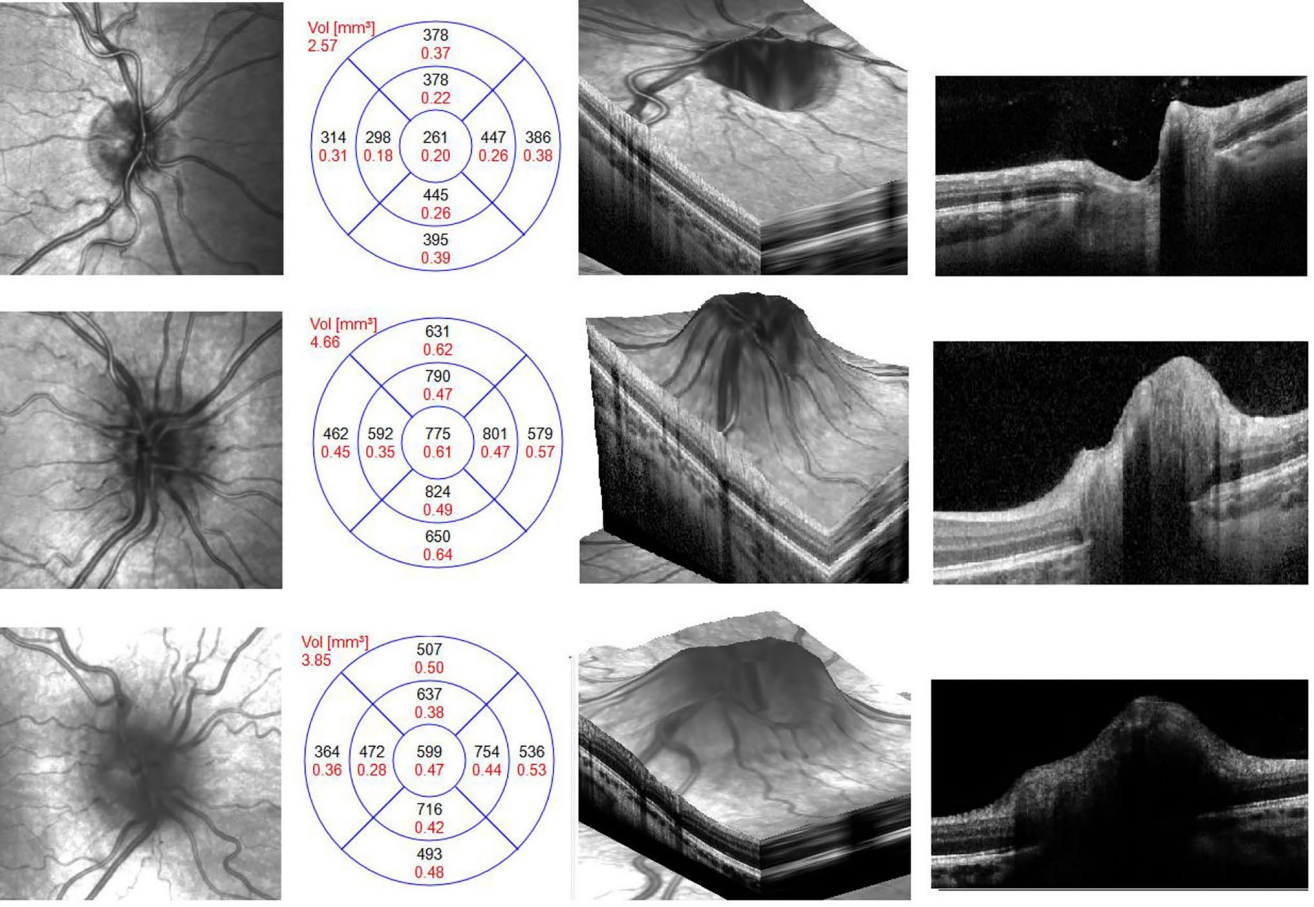
OCT images of 3 patients with sagittal synostosis. Columns show OCT image of the optic nerve disc (1), corresponding peripapillary total retinal thickness values (μm, in black) and total retinal volume values (mm^3^, in red) for each quadrant (2), OCT 3-D reconstruction images of the optic disc (3) and cross-sectional images of the total retinal thickness (4). In patient 1, fundoscopy appearance and OCT values are normal, in patient 2, fundoscopy is normal while OCT values are abnormal, and in patient 3, papilledema is seen on fundoscopy and OCT parameters are increased

When applying the above-established cut-off points to the sagittal synostosis group, TRT is found to be increased in 16 of the 93 patients (17%) and TRV in 15 of the 93 patients (16%). In 15 cases with increased TRT, the TRV was increased as well. Furthermore, both OCT parameters were abnormal in 3 out of the 4 patients with papilledema. Both TRT and TRV showed an association with appearance on fundoscopy (OR = 16.7, 95% CI 1.71–458.2, *p* = 0.02, and OR = 18.2, 95% CI 1.86–502.79, *p* = 0.01, Fisher’s exact test) (Table 3). A decreased TRT suspected of atrophy was not seen in this cohort. OCT images of patients with and without papilledema are shown in Fig. 4.

**Table 3:** Association between OCT and fundoscopy in sagittal synostosis patients

	No papilledema	Papilledema	Total	OR	95% CI	*p*
TRT						
Normal Increased Total	76 13 89	1 3 4	77 16 93	16.7	1.71–458.21	**0.02**
TRV						
Normal Increased Total	77 12 89	1 3 4	78 15 93	18.2	1.86–502.79	**0.01**

The eye with the highest peripapillary TRT was chosen (*n* = 93)

*TRT* total retinal thickness, *TRV* total retinal volume, *OR* odds ratio, *CI* confidence interval

From the 16 sagittal synostosis patients with increased TRT, two patients were diagnosed with pseudopapilledema (+ 5D and + 8D), which explains the abnormal values, and were therefore excluded from additional analyses. One of the latter patients had a normal TRV (Fig. 4).

The presence of clinical signs suggestive of ICH in patients with abnormal OCT parameters is presented in Table 4. Skull growth arrest was significantly associated with increased TRT and TRV.

**Table 4:** Association between abnormal OCT parameters (TRT and TRV) and clinical signs of ICH in sagittal synostosis patients

	**OCT normal *N* = 77**	**OCT abnormal *N* = 14**	**Total**	**OR**	**95% CI**	***p***
Skull growth arrest	2/74 (3%)	4/14 (29%)	88	13.65	2.25–113.47	** < 0.01**
Fingerprinting	11/48 (23%)	4/7 (57%)	55	4.34	0.82–25.22	0.08
Papilledema	1/77 (1%)	3/14 (21%)	91	19.53	1.98–541.40	**0.01**
Headache	4/57 (7%)	3/11 (27%)	68	4.80	0.81–26.41	0.08

Two patients diagnosed with pseudopapilledema were excluded from analyses (*N* = 91). The eye with the highest peripapillary TRT was chosen. In case of an abnormal OCT, both parameters (TRT and TRV) were increased

*TRT* total retinal thickness, *TRV* total retinal volume, *OR* odds ratio, *CI* confidence interval

### Sagittal synostosis cohort: differences in quadrants

With respect to TRT, abnormal values of the outer peripapillary ring and inner peripapillary ring were reported in 12% (10/84) and 17% (15/89) of the sagittal synostosis patients, respectively (Table 5). There are indications that both are associated with the occurrence of papilledema (Fisher’s exact test OR 28.55, 95% CI 2.79–816.98, *p* < 0.01 and OR 17.28, 95% CI 1.76–477.00, *p* = 0.01, respectively, for both TRT and TRV). With regard to the different quadrants of the TRT, the superior, nasal, and inferior quadrant were found to be associated with papilledema, the highest OR is found in the nasal quadrant (Fisher’s exact test OR 13.71, 95% CI 1.41–375.77, *p* = 0.02, OR 97.51, 95% CI 7.91–3310.14, *p* < 0.01, and OR 35.9, 95% CI 3.47–1042.97, *p* < 0.01, respectively). Similar results are found for TRV, except for the nasal quadrant (OR 69.31, 95% CI 6.06–2184.54, *p* < 0.01).

**Table 5:** Association between papilledema and inner, outer peripapillary ring, and different quadrants of the peripapillary TRT in sagittal synostosis patients

	No papilledema	Papilledema	Total	OR	95% CI	*p*
Outer ring						
Normal Increased Total	73 7 80	1 3 4	74 10 84	28.55	2.79–816.98	** < 0.01**
Inner ring						
Normal Increased Total	73 12 85	1 3 4	74 15 89	17.28	1.76–477.00	**0.01**
Superior quadrant						
Normal Increased Total	67 14 81	1 3 4	68 17 85	13.71	1.41–375.77	**0.02**
Nasal quadrant						
Normal Increased Total	85 2 87	1 3 4	86 5 91	97.51	7.91–3310.14	** < 0.01**
Inferior quadrant						
Normal Increased Total	80 6 86	1 3 4	81 9 90	35.96	3.47–1042.97	** < 0.01**
Temporal quadrant						
Normal Increased Total	83 4 85	3 1 4	86 5 91	6.11	0.22–77.19	0.21

Two patients diagnosed with pseudopapilledema were excluded from analyses (*N* = 91). The eye with the highest TRT was chosen. Similar results are found for the TRV, except for the nasal quadrant (OR 69.31, 95% CI 6.06–2184.54, *p* < 0.01)

*OR* odds ratio, *CI* confidence interval, *TRT* total retinal thickness, *TRV* total retinal volume

### Sagittal synostosis cohort: follow-up of patients with a normal OCT scan

From the 77 sagittal synostosis patients with a normal OCT scan, three patients were suspected of ICH. One patient with skull growth arrest and a stable Chiari malformation on MRI had papilledema on fundoscopy which resolved after 4 months without treatment. The patient is under close surveillance. In two patients, ICH was confirmed on invasive ICP monitoring, whereas fundoscopy and OCT were normal. The first patient presented with frequent headache and elevated ICP 1 month prior to the OCT and underwent surgery. The second patient was a late referral who presented with neck pain, development problems, and a Chiari malformation on MRI. Primary surgery was performed.

Furthermore, ICP measurement revealed normal pressures in 4 patients with normal fundoscopy and OCT values. Indications for ICP monitoring were frequent headache in 2 patients, headache in combination with skull growth arrest and sleeping problems in one, and sleeping problems in combination with fingerprinting on skull X-ray in the other patient.

## Discussion

This study applied OCT measurements across a large sample of a single type of non-syndromic craniosynostosis. Three findings arise from this study. First, this study provides cut-off points for TRT and TRV obtained automatically with SD-OCT, which can be easily applied in the clinic to make comparisons with values in children with optic nerve diseases. Second, using our normative references, we found a correlation between abnormal OCT parameters and presence of papilledema on fundoscopy in sagittal synostosis patients. Third, the present study detected abnormalities with OCT more often than with fundoscopy (17% on OCT vs 4% on fundoscopy), which were associated with skull growth arrest and are suspected for ICH.

Our study shows that there was no significant correlation of gender and age on TRT nor TRV. This is in line with pediatric normative reference studies by Turk et al. Yanni et al. and Rotruck et al. [24–26], where gender and age were not correlated to RNFL/TRT. The normal values can therefore be applied in the sagittal synostosis cohort despite the fact that this population mainly consists of males.

We found an association between abnormal OCT parameters and papilledema on fundoscopy. An increased RNFL in response to ICH detected with direct intraoperative intracranial pressure measurement in craniosynostosis was reported by Swanson et al. [8]. A study by Vartin et al. [9] on patients with idiopathic intracranial hypertension found an association for both RNFL and TRT with elevated ICP. The association between increased OCT parameters and papilledema on fundoscopy indicates that papilledema as a sign of ICH is an objectifiable phenomenon consistent with the current opinion that the presence of papilledema is a strong indicator of ICH. However, the absence does not exclude ICH.

In this study, OCT identified more abnormalities compared to fundoscopy (17 vs 4%). This is in line with the study of Swanson et al. [8], who reported a higher percentage of OCT abnormalities compared to fundoscopy in patients with craniosynostosis. Sensitivity for detecting invasively measured ICH was superior for OCT compared to fundoscopy (89% vs 11%). Based on the objectivity of OCT, it might be expected that changes in the retina due to ICH are accurately recognized by OCT and rates presented by fundoscopy are an underestimation, as suggested by Wall et al. [27] and Thomas et al. [28]. Both studies performed invasive ICP monitoring pre- and/or postoperatively in patients with sagittal synostosis and reported increased rates of ICH compared to studies in which ICH was based on fundoscopic examination.

Moreover, in our study, clinical signs of ICH were more prevalent among patients with abnormal OCT parameters. Development of ICH during follow-up in isolated sagittal synostosis patients seems related to reduced ICV in particular. OFC has shown to be a reliable indicator of ICV [14, 29] and is associated with the occurrence of headache in patients with sagittal synostosis [30]. Skull growth arrest has shown to be correlated with the development of papilledema in sagittal synostosis [31]. Therefore, abnormalities detected with OCT are associated with skull growth arrest and thus certainly suspected for ICH.

Furthermore, abnormalities detected on OCT might also reflect the normalization process of the retina in patients with a history of ICH. The time after which retinal changes normalizes is still unknown. Therefore, OCT is not only of added value in detecting ICH but also during individual follow-up, which prompts closer monitoring (and earlier action in case of signs of ICH).

Also, one could hypothesize that, after exluding pseudopapilledema, an increase within the reference values during follow-up can be a sign of ICH. Swanson et al. and Kalmer et al. [8, 32] suggest that borderline ICP can also lead to abnormalities of the retina. However, longitudinal studies should determine the outcome of patients with abnormal OCT parameters, the time after which retinal changes normalize, and the meaning of an increase of TRT within the reference values.

In addition, as suggested by Fard et al. [33], TRV might be succesful in differentiating papilledema from pseudopapilledema. In the present study, one patient with pseudopapilledema had an increased TRT and normal TRV. Further research should determine the value of TRV for distinction of papilledema from pseudopapilledema.

However, we have to keep in mind that OCT measures are indirect markers of ICH. In this study, 2 patients did not develop papilledema on fundoscopy or OCT while elevated intracranial pressures were found. The exact mechanism is still unknown, but it has been suggested that the development of retinal changes might be influenced by anatomic factors, such as the distensibility of the intraocular optic nerve support and axonal elements, the optic nerve canal, or ocular pressure [8, 34–37].

### Clinical impact

The present study shows that increased TRT and TRV are highly suspicious of ICH and associated with skull growth arrest. Based on these findings, we recommend screening for ICH in sagittal synostosis using OCT, fundoscopy, and skull growth measurements until the age of 6 years. Clinical symptoms and an abnormal OCT scan and/or fundoscopy after excluding pseudopapilledema is highly suggestive of ICH. The combination of a normal OCT and fundoscopy does not exclude ICH. Therefore, ICP measurement should be considered in patients with clinical symptoms of ICH such as skull growth arrest with normal fundoscopy and OCT.

One limitation of our study is the small groups of positive cases suspected of ICH. Due to the low number of positive cases, it was not possible to draw conclusions with great certainty as the results contained large confidence intervals.

Also the healthy cohort comprised a small sample size. Besides, patients in the sagittal synostosis group were significantly younger compared to the healthy children from which the normative references were derived. Furthermore, axial length of the globe was not measured in both cohorts. To minimize any potential error caused by very high or low axial lengths, we excluded ≥ + 4.00D and ≤ − 2.5D in keeping with normative databases for OCT devices. Unfortunately, RE was not measured in the sagittal synostosis cohort. Although mild refractive errors did not affect the OCT parameters in the control cohort, high hyperopia (+ 5 and + 8 diopters) in two patients with sagittal synostosis caused an increase in TRT. Therefore, as with fundoscopy, care must be taken when utilizing OCT to detect ICH in patients with high refractive errors.

Also, we did not have 24-h overnight ICP measurements in all patients. The definition of ICH remains a challenge. The gold standard is the 24-h ICP measurement, although objective cut-off values for children of various ages are lacking. Given the invasive nature of the direct ICP measurement, it is only used in selected cases. In lack of ICP measurements, we tested the value of OCT as screening tool, using cut-off values from a healthy cohort of children, and compared OCT to the most used screening tool fundoscopy. As presence of papilledema is not sufficient to screen for ICH solely because of the false-negative results, we used several other clinical parameters, such as skull growth arrest as a proxy of ICH, which reduces the chance of missing ICH.

There is also a debate regarding fingerprinting as a clinical sign of ICH. Several studies report a low sensitiviy to detect ICH [38–42]. However, a recent study has shown that fingerprinting resolves after surgical correction for ICH. Therefore, the appearance of diffuse fingerprinting might suggest ICH, although clinical decisions cannot be based solely on the presence of fingerprinting. In addition, Zifel et al. [20] showed an association with the intracranial reserve capacity, indicating the reserve capacity is already exhausted in case of severe fingerprinting on skull X-rays. To minimize false-positive results in our study, we only included patients with severe diffuse fingerprinting. In future studies, machine learning could be used as an objective and effective tool to quantify and score skull results. Thereby the occurrence or progression of fingerprinting should be taken as a proxy instead of the observation based on one skull X-ray(fingerprinting yes/no).

In conclusion, our established cut-off points can be applied to identify ICH in pediatrics. OCT has the potential to detect patients suspected of ICH, which are not found with fundoscopy. Furthermore, skull growth arrest is associated with increased OCT parameters and therefore is a relevant clinical measure that should raise alertness. OCT combined with other clinical symptoms can be of added value in the clinical decision-making in craniosynostosis.
